# Study of Channel Characteristics for Galvanic-Type Intra-Body Communication Based on a Transfer Function from a Quasi-Static Field Model

**DOI:** 10.3390/s121216433

**Published:** 2012-11-27

**Authors:** Xi Mei Chen, Peng Un Mak, Sio Hang Pun, Yue Ming Gao, Chan-Tong Lam, Mang I. Vai, Min Du

**Affiliations:** 1 Department of Electrical and Computer Engineering, Faculty of Science and Technology, University of Macau, Macau 999078, China; 2 Laboratory of Medical Instrumentation and Pharmaceutical Technology of Fujian Province, Fuzhou 350108, China; 3 State Key Laboratory of Analog and Mixed-Signal VLSI, Faculty of Science and Technology, University of Macau, Macau 999078, China; 4 College of Physics and Telecommunication Engineering, Fuzhou University, Fuzhou 350108, China; 5 Macao Polytechnic Institute, Macau 999078, China

**Keywords:** Body Sensor Network (BSN), BER, channel characteristics, Intra-Body Communication (IBC), modulation scheme, quasi-static field model, transfer function

## Abstract

Intra-Body Communication (IBC), which modulates ionic currents over the human body as the communication medium, offers a low power and reliable signal transmission method for information exchange across the body. This paper first briefly reviews the quasi-static electromagnetic (EM) field modeling for a galvanic-type IBC human limb operating below 1 MHz and obtains the corresponding transfer function with correction factor using minimum mean square error (MMSE) technique. Then, the IBC channel characteristics are studied through the comparison between theoretical calculations via this transfer function and experimental measurements in both frequency domain and time domain. High pass characteristics are obtained in the channel gain analysis *versus* different transmission distances. In addition, harmonic distortions are analyzed in both baseband and passband transmissions for square input waves. The experimental results are consistent with the calculation results from the transfer function with correction factor. Furthermore, we also explore both theoretical and simulation results for the bit-error-rate (BER) performance of several common modulation schemes in the IBC system with a carrier frequency of 500 kHz. It is found that the theoretical results are in good agreement with the simulation results.

## 1. Introduction

Recently, Body Area Networks (BANs) have been developed to facilitate low power devices operating on, in or around the human body to serve a variety of applications including medical and consumer electronics [1]. After intensive research for decades, wearable electronics for personal health care in BANs have been migrating from the research arena to applications [2]. As one of the foundational building blocks in BANs, the physical layer should be reliable, with low power consumption, and highly secure, but with relatively less demand in data rate requirements [3]. Currently, there are many potential candidates for the physical layer of BANs, such as Zigbee, Bluetooth, Ultra Wide Band (UWB) and Intra Body Communication (IBC) [3,4]. The most commonly used low power, short range technologies belong to the radio wave types, although they were originally not optimized for devices operating on or inside the human body. To serve as a convenient physical layer of BANs, galvanic-type IBC has been attempting to become a front-runner candidate. However, little is known about its communication channel characteristics, such as transfer function, harmonic distortion, modulation scheme, and so on.

Galvanic-type IBC is a relatively new communication technology using the human body as a channel for data communication. The transmitter converts the transmitted signal into a flow of ionic current within the tissue and the receiver, which is located somewhere on the body, recovers the original signal information by detecting the ionic current flow. The low ionic current conveys the signal successfully and no obvious local body heating [5] is observed. The measured transmitted power consumption can be as low as 8 μW [6] for sending an ECG signal from thorax to the wrist. The cadaver experiments of Lindsey *et al.*[7] and the *in vitro* experiment of Wegmueller *et al.*[8] also showed that IBC is able to effectively deliver signal for implants. Since IBC is not a radiation methodology, low frequency carrier (less than 1 MHz) is a possible and common selection. The advantages of using low frequency carrier, in general, can minimize the local heating [9], and allow one to simplify the design of the transceiver, thus reducing the overall power consumption (system clock) and the risk of eavesdropping at the expenses of data rate. Fortunately, the data rate requirement for home-based healthcare data monitoring can be relative low, e.g., 6 kbps in ECG, 7.2 kbps in SpO2 and 2.4 bps in body temperature surveillance [10]. Therefore, the galvanic-type IBC could be a choice to build a medical data monitoring system in a BAN. As a matter of fact, we have witnessed a couple of prototype devices [8,11,12] that were designed based on the galvanic-type IBC in recent years.

The issue of utilizing an analytical model to study channel characteristics plays an important role in both understanding signal transmission mechanism and hence later for IBC system design. With the purpose of obtaining channel characteristic, several IBC channel models have been proposed in the recent literature for the two general coupling-types of IBC, namely galvanic-type and capacitive-type. Generally, galvanic-type IBC [6] transmits signals within the human body primarily via two pairs of on-body electrodes while a capacitive-type IBC [13] needs only two electrodes on the body and a return path formed by a nearby coupling environment; hence being more environment dependent. The galvanic-type IBC usually operates at lower frequency than the capacitive-type but suffers less interference with the trade-off of higher attenuation. For capacitive-type IBCs, since they are generally operated in higher frequency, traditional electromagnetic (EM) analysis techniques are employed, especially by various numerical simulation methods. For examples, model [14] used the finite difference time domain (FDTD) technique and model [15] used the finite element (FEM) technique to numerically study the received field signals. So far, very few EM analytical models for IBC were published. For example, Sasamori *et al.*[16] derived the high-frequency (around 2.45 GHz) asymptotic representation via the scattered EM field analysis. However, it was targeted for EM field propagation, not for data communication. Other literatures have proposed easier alternative circuit models (either distributed or lumped circuits) to describe IBC channels. Cho *et al.*[17] described one of the earliest distributed RC models to study the capacitive-type IBC channel and the relationship between received power and transmitted power was empirically obtained with little physical meanings. Hachisuka *et al.*[18] were one of the earliest groups to study both galvanic and capacitive-type IBC channels. However, their four-terminal circuit model is too simple and needs more refinements. For a galvanic-type IBC, Wegmueller *et al.*[19] obtained an entire transfer function based on the improved simple four-terminal circuit model including the coupling electrode impedances using a Cole-Cole reference model. Nevertheless, their model did not contain much underlying principle explanation or the discussion of communication schemes. Recently, Song *et al.*[20] improved the circuit model of [19] by adding more circuit impedances (such as internal resistances of the IBC devices, human body impedances calculated from the electrical property of tissues and *etc.*) to come up with a simulation method for a galvanic-type IBC model, which requires a subject-dependent and region-dependent correction factor to compensate for a typical deviations of 20 dB between the simulation results and experimental values. Recently, Callejon *et al.*[21,22] developed asymmetric distributed circuit models for galvanic-type and capacitive-type IBCs and obtained a transfer function resembling basic transmission line techniques. However, the model only considered skin attenuation and dispersion character but neglected all other human tissues (such as muscle, *etc.*). Hence, further researches into the time domain characteristic and communication performance are needed to be conducted for the above models. Especially, the channel characteristic for an IBC channel has not yet fully studied (*i.e.*, transfer function below 1 MHz, harmonic distortion, *etc.*).

Our work here concentrates on the galvanic-type IBC because of its secure and lesser interference features. Specifically, we based it on the analytical quasi-static EM model from our group in [23] for the IBC signal distribution within a human limb, including the effect of most tissues (skin, fat, muscle, and bone). Then we extract an analytical communication channel system transfer function from a quasi-static field model to study both frequency domain and time domain characteristics with validations from experiments; and also to examine the communication performance for different modulation schemes. As in any other models, imperfections do exist due to many inherent factors (such as irregular human shapes and variability in human tissue parameters due to human physiology during various metabolisms). Therefore, a subject dependent correction factor was semi-empirically introduced first into the transfer function to compensate for the inadequacies of original model. Subsequent analyses (channel gain, harmonic distortion, communication schemes, *etc.*) using this corrected transfer function were obtained through calculation. Then the calculation results were compared with the experimental studies. Also, we studied suitable modulation schemes for the IBC channel through theoretical calculation and simulation via the corrected model.

The arrangement of this article is as follows: Section 2 briefly reviews the quasi-static field model of a galvanic-type IBC in order to obtain the channel system transfer function up to 1 MHz and also improve the transfer function model semi-empirically by the MMSE technique; Section 3 investigates the channel characteristics in the time domain via pulse response in both baseband and passband transmissions; Section 4 evaluates different possible modulation schemes over IBC. Finally, the conclusions of this paper can be found in Section 5.

## 2. Transfer Function of Galvanic-Type IBC

### 2.1. Transfer Function Frequency Response Based on Quasi-Static Field IBC Model

**Figure 1.**
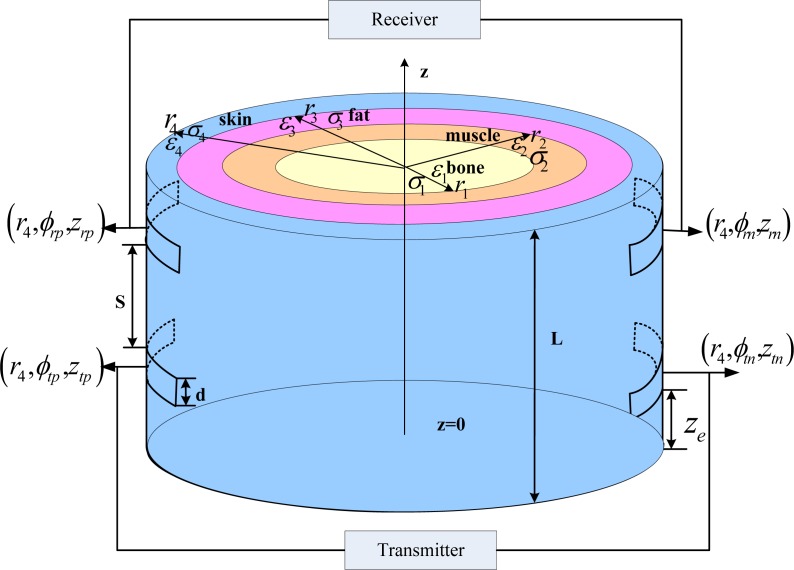
Illustration of galvanic-type IBC geometry on the human limb.

Based on our previous work [23], we use two pairs of electrodes to transmit and receive electrical signals across the human body in the galvanic-IBC model. As depicted in Figure 1, the human limb can be represented by a length of *L* with concentric cylinder layers containing the skin, fat, muscle and bone tissues with radii (*r*_4_, *r*_3_, *r*_2_, *r*_1_), respectively. The electrical properties of each tissue layer are assumed to be homogeneous and characterized by permittivities (*ε*_4_, *ε*_3_, *ε*_2_, *ε*_1_) and conductivities (*σ*_4_, *σ*_3_, *σ*_2_, *σ*_1_). From [23], as the frequency falls below 1 MHz, the inductive effect, propagation effect and radiation from the skin into air can be neglected, while the capacitance effect cannot be ignored. Applying the quasi-static approximation [24] to the Maxwell’s Equations, the governing equation of electric potential in cylindrical coordinate system can be simplified into Equation (1) below:

(1) $$\nabla \cdot \sigma_{\mathrm{cs}}(f)\nabla V\approx 0\;s=1\ldots 4$$

where *V* represents the electric potential within the human limb, *σ_cs_*(*f*) is composite conductivity of layer *s* at frequency *f*, and:

(2) $$\sigma_{\mathrm{cs}}(f)=\sigma_{s}(f)+j2\pi f\varepsilon_{\mathrm{rs}}(f)\varepsilon_{0}$$

Note that *σ_s_*(*f*) and *ε_rs_*(*f*) are the conductivity and relative permittivity of layer *s* at frequency *f*, respectively. *ε_o_* is the permittivity of free space. Considering with proper electromagnetic boundary conditions, the electric potential distribution on the surface (*r_4_*, *ϕ*, *z*) of the human limb will be:

(3) $$\begin{array}{lll} V_{4}(f,r_{4},\varphi ,z) & = & \sum_{k=1}^{\infty }\sum_{n=1}^{\infty }[E_{4\mathrm{kn}}(f)I_{n}\left(\frac{k\pi }{L}r_{4}\right)\text{cos}(n\varphi )+F_{4\mathrm{kn}}(f)I_{n}\left(\frac{k\pi }{L}r_{4}\right)\text{sin}(n\varphi )\\ & & +G_{4\mathrm{kn}}(f)K_{n}\left(\frac{k\pi }{L}r_{4}\right)\text{cos}(n\varphi )+H_{4\mathrm{kn}}(f)K_{n}\left(\frac{k\pi }{L}r_{4}\right)\text{sin}(n\varphi )]\cdot \text{sin}\left(\frac{k\pi }{L}z\right) \end{array}$$

where *I_n_* is the modified Bessel function of the first kind of order *n*, *K_n_* is the modified Bessel function of second kind of order *n*, *E*_4*kn*_(*f*), *F*_4*kn*_(*f*), *G*_4*kn*_(*f*) and *H*_4*kn*_(*f*) are the parameters on layer *s* = 4 at frequency *f* and can be solved by the method stated in [23].

Using Equation (3), the transfer function of the galvanic-type IBC can be obtained. We assume that ideal electrodes (the thickness of electrode, the impedance between electrode and skin are negligible) are employed for the transmitter and receiver sites. The corresponding electric potential can then be represented by the value at the center of the electrode, and the output differential potential at the receiver can be found as:

(4) $$Y(f)=V_{4}(f,r_{4},\varphi_{\mathrm{rp}},z_{\mathrm{rp}})-V_{4}(f,r_{4},\varphi_{\mathrm{rn}},z_{\mathrm{rn}})$$

where *V*_4_(*f, r*_4_, *ϕ_rp_, z_rp_*) and *V*_4_(*f, r*_4_, *ϕ_rn_, z_rn_*) are the electric potentials at frequency *f* at the positive and negative electrode of receiver with locations of (*r*_4_, *ϕ_rp_, z_rp_*) and (*r*_4_, *ϕ_rn_, z_rn_*) respectively.

Similarly, the applied differential signal at the transmitter side of galvanic-type IBC channel can be expressed as:

(5) $$X(f)=V_{4}(f,r_{4},\varphi_{\mathrm{tp}},z_{\mathrm{tp}})-V_{4}(f,r_{4},\varphi_{\mathrm{tn}},z_{\mathrm{tn}})$$

where *V*_4_(*f, r*_4_, *ϕ_tp_, z_tp_*) and *V*_4_(*f, r*_4_, *ϕ_tn_, z_tn_*) are the electric potentials at the positive and negative electrode of transmitter site, respectively.

Using Equations (4) and (5), the transfer function of the Galvanic-type IBC can be found as:

(6) $$H(f)=\frac{V_{4}(f,r_{4},\varphi_{\mathrm{rp}},z_{\mathrm{rp}})-V_{4}(f,r_{4},\varphi_{\mathrm{rn}},z_{\mathrm{rn}})}{V_{4}(f,r_{4},\varphi_{\mathrm{tp}},z_{\mathrm{tp}})-V_{4}(f,r_{4},\varphi_{\mathrm{tn}},z_{\mathrm{tn}})}\;1\mathrm{Hz}\leq f\leq 1\mathrm{MHz}$$

Equation (6) is a complex function of both electrical properties and geometry of tissue layers. The numerical value of Equation (6) can be calculated using the human tissue parameters from Gabriel *et al.*[25], together with typical approximate human limb tissue geometries from the individual person. For safety purposes, in our experiment we have used electrical stimulation square type electrodes with dimensions of 40 mm × 40 mm. For simplicity, the impedance of the electrode was omitted in the later calculation as the measured impedance of the stimulating electrode excluding the tissue is about 40 Ω, which is much lower than the human limb impedance of around several hundred Ohms [26]. The input signal was biphasic waveform with limitation of peak value 1 mA in order to comply with the safety standard [27] during *in vivo* experiments. The mathematical expression of the input signal was modeled as the following uniform normal current density:

$$J_{n}(r_{4},\varphi ,z)=\{\begin{array}{c} 1\mathrm{mA}/S_{e}\;z_{e}\leq z<z_{e}+d\;\mathrm{and}\;0\leq \varphi <1.23\mathrm{rad}\\ -1\mathrm{mA}/S_{e}\;z_{e}\leq z<z_{e}+d\;\mathrm{and}\;3.14\mathrm{rad}\leq \varphi <4.37\mathrm{rad} \end{array}$$

where *S_e_* is the area of the electrode; *z_e_* = 70 mm and *d* = 40 mm (shown in Figure 1) represent the corresponding position and size of the electrodes.

**Figure 2.**
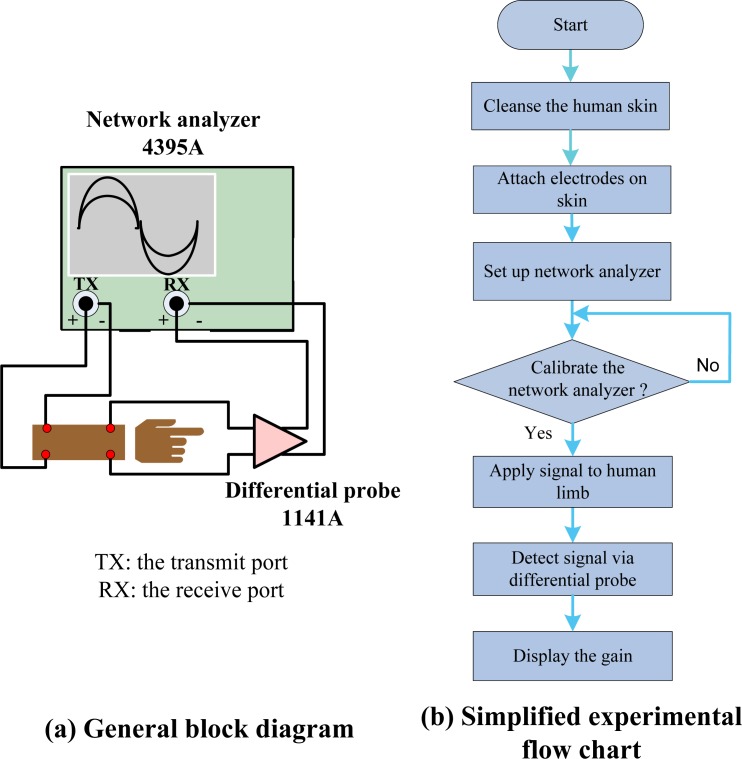
*In-vivo* IBC channel gain experiment: (**a**) General block diagram and (**b**) Simplified experimental flow chart.

The calculated results were then compared with *in vivo* experiments. A network analyzer (Agilent, 4395A Network/Spectrum/Impedance Analyzer) was used to take measurements from 100 Hz to 1 MHz. Several young healthy adult subjects were recruited for our *in vivo* experiments. Without loss of generality, two typical sets of data were shown in this article [Subject A: male, height 1.85 m, weight 90 kg, Body Mass Index (BMI) 26.01, length of lower arm 280 mm and Subject B: female, height 1.57 m, weight 50 kg, BMI 20.2, length of lower arm 250 mm]. In the model calculation, the radius of limb for subject A was proximately 35 mm, with the skin, fat, muscle and bone thicknesses of 3 mm, 4 mm, 11 mm and 17 mm [28], respectively; while the counterparts for subject B were 30 mm, 2.5 mm, 3.4 mm, 9.4 mm and 14.7 mm. The *in vivo* experiment set up, which was composed of a network analyzer and differential active probe (Agilent, 1141A Differential Probe), is shown in Figure 2(a) while the corresponding flow chart is displayed in Figure 2(b). Also the input impedance matching issues [29] related to changes of body electrical parameter can be ignored in Figure 1 because of our high input impedance (1 MΩ) of differential probe and operation of sub-MHz low frequency region. Then, the high input impedance bio-amplifier is utilized to extract the conduction current for further processing. Prior to the experiments, the skins of the subjects were cleansed to make good contact with the electrodes. The sinusoidal signal of different frequencies (100 Hz–1 MHz) from the output port of calibrated network analyzer was applied to the human limb at the transmitter sites, and the received signal at receiver sites was detected via the differential active probe. Then gain *versus* frequency was obtained in network analyzer by sweeping the frequency. For each subject, both calculation and experimental results of two separation distances (S = 6 cm and 11 cm) between transmitter and receiver are reported here.

**Figure 3.**
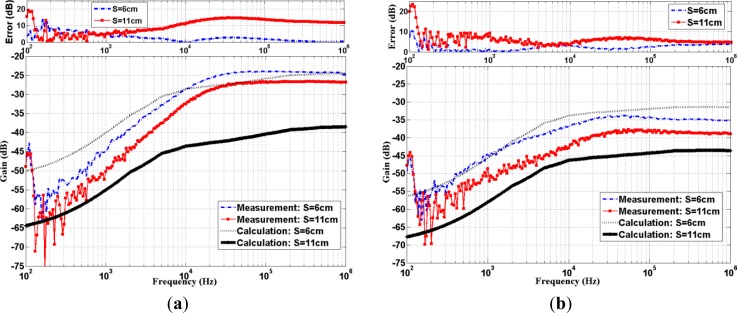
Calculated and measured transfer function characteristic of (**a**) Subject A; (**b**) Subject B.

Both calculated and measured results of IBC channel gain are shown in Figure 3. In addition, their absolute errors between the calculation and measurement over the frequency range (100 Hz to 1 MHz) are shown at the top in Figure 3. One can observe that our theoretical and measured IBC channel exhibits high pass characteristics. The passband starts at around 20 kHz with their maximum error below 16 dB. At S = 6 cm, measurement gains are lower than −25 dB (and −35 dB) below 20 kHz, then sustains around −25 dB (and −35 dB) of relative-flat gain for the frequency higher than 20 kHz for subject A (and subject B), respectively. In addition, the measurement gain decreases several dB (3–6 dB) for two subjects as the transmission distance increases additional 5 cm (*i.e.*, S = 11 cm). Note that the low attenuation region happened to be over 20 kHz and the measurement flat response at high frequency region is improved over results of the reference [23] by the usage of newly acquired differential active probe.

### 2.2. Transfer Function with Correction Factor

Even though both the calculated and measured results of the IBC channel exhibit similar high pass characteristics, the calculated values from Equation (6) obviously deviate somewhat from the measurements. These sources of error are intrinsic and can result from several aspects. The first obvious reason for deviations comes from the fact that the actual shapes of all subjects’ limbs vary somewhat from our concentric cylindrical model. Another major reason is due to the uncertainties of electrical properties in our *in vivo* subjects’ tissues. In the galvanic-type IBC, the signal mainly flows within the human body, especially with low frequency carrier. The electrical properties of the tissues significantly influence the propagation of the signal. From the literatures, the physiological status variations of the tissues induce fluctuations in electrical properties [30]. Additionally, in accordance to the experiment procedures of Gabriel *et al.*[31], the measurements have ±(15–25)% uncertainties at low frequencies due to physiological processes and there were three sources of materials, including human autopsy materials and sheep [31]. From the top of Figure 3, one can observe that obvious variations happen at low frequencies (<1 kHz), which is caused by aforementioned effect and/or other nearby artifacts there. Owing to the larger attenuation and fluctuation there, this frequency band would not be used in IBC signal transmission. Hence, we will focus our investigation between 1 kHz to 1 MHz for the rest of this paper.

In order to take into account of non-avoidable variations, a correction factor *A*(*f*) is introduced to Equation (6) in order to compensate the variation effects. Hence, the transfer function of the IBC would become:

(7) $$H_{\Pi }(f)=A(f)\cdot \frac{V_{4}(f,r_{4},\varphi_{\mathrm{rp}},z_{\mathrm{rp}})-V_{4}(f,r_{4},\varphi_{\mathrm{rn}},z_{\mathrm{rn}})}{V_{4}(f,r_{4},\varphi_{\mathrm{tp}},z_{\mathrm{tp}})-V_{4}(f,r_{4},\varphi_{\mathrm{tn}},z_{\mathrm{tn}})}$$

where *A*(*f*) is the semi-empirical correction factor, related to the individual human body. Note *A*(*f*) corrects the inadequacies of the uncompensated model that did not account for individual subject characteristic. As seen in Figure 3, both the calculation and measurement results possess the similar trend, their difference is approximately constant. This is especially true in our frequency of interest (*i.e.*, 20 kHz–1 MHz), for which both experimental and calculation results are relatively flat. Hence, for the ease of handling at this stage to determine the correction factor, we assumed the value of *A*(*f*) is independent of frequency *f* (denoted as *A*). Alternatively, the transfer function in dB is given as:

(8) $$H_{\Pi \mathrm{dB}}(f)=20\cdot {\text{log}}_{10}(|H(f)|)+K$$

where *K =* 20log_10_(*|A|*). The determination of correction factor *A* in Equation (7) is equivalent to determining the parameter *K* in Equation (8). Note that *K* is subject-dependent to compensate the variations of tissues’ parameters in different subjects. In order to find K numerically, we replace *K* with variable *k*, and have:

(9) $$H_{\Pi \mathrm{dB}}(f,k)=20\cdot {\text{log}}_{10}(|H(f)|)+k$$

Then *K* can be found by minimizing the mean square error (MMSE) between the calculated result in Equation (9) and the measurements over the less fluctuation frequency band (1 kHz to 1 MHz):

(10) $$K=\underset{k}{\text{arg}\;\text{min}}E\left[{\left(\left|{\hat{H}}_{\mathrm{dB}}(f)-H_{\Pi \mathrm{dB}}(f,k)\right|\right)}^{2}\right]$$

where *H*_Π*dB*_(*f,k*) represents calculation value for the individual person plus the value *k*. And *Ĥ_dB_*(*f*) is the measurement value for the subject.

**Figure 4.**
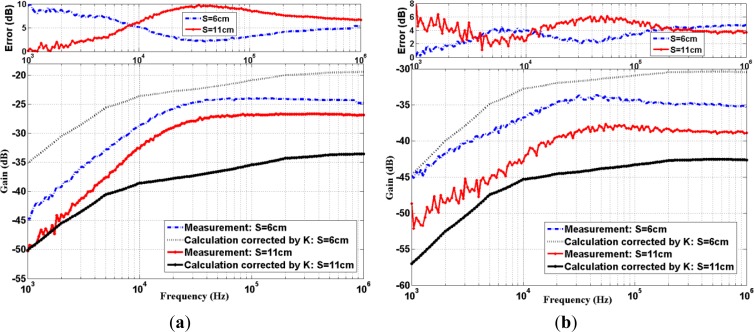
Measured and calculated (with correction) transfer function characteristic of (**a**) subject A; (**b**) subject B.

With the introduction of the constant correction factor *K* in Equation (8), the transfer function is then re-evaluated. By solving Equation (10), *K* is found to be around +5.5 dB and +1.5 dB for subject A and subject B, respectively. The comparisons between the calculation results using corrected models and the experimental results can be found in Figure 4. Again, their absolute errors are shown at the top of the figure. One can find that the model with correction factor *K* matches well with the experimental. Numerically, the maximum absolute error is generally lower than 10 dB over the useful band of low attenuation transmission frequencies (*i.e.*, 20 kHz to 1 MHz), *i.e.*, ultra-sound up to validity frequency (1 MHz) of quasi-static field model. This, in turn, would demonstrate the validity that a simple constant *K* factor will suffice for our model improvement here.

## 3. IBC Time Domain Responses Based on Corrected Transfer Function

Due to the frequency dependence of the transfer function (7), a transmitted signal would suffer dispersion and amplitude reduction. In digital communication, this phenomenon will cause pulse spreading and overlapping in the transmitted pulses. Hence, this will hinder the data communication rate. For this reason, both baseband and passband transmissions have been investigated by transmitting square waves into the IBC channel to assess the channel dispersion and distortion behavior parameters, which could assist in IBC design. In this Section, we study the IBC time domain channel characteristics with the corrected analytical model discussed in Section 2.2. For simplicity, only Subject B calculation and measurement results are shown throughout this Section.

**Figure 5.**
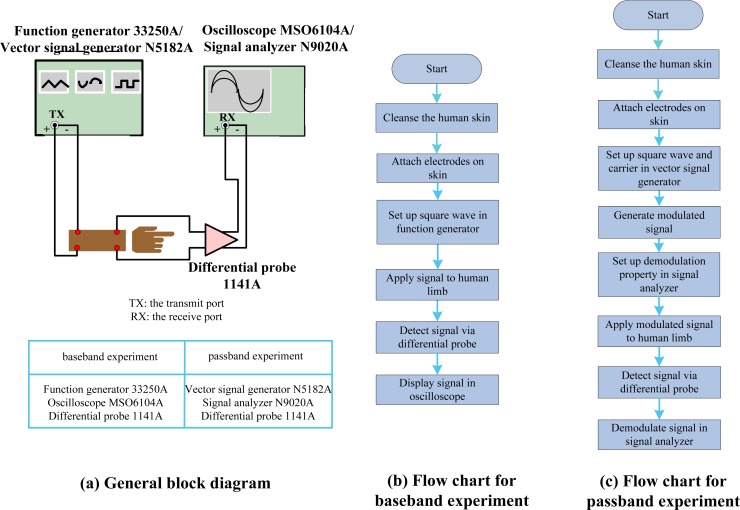
IBC channel baseband and passband transmission experiment set-ups: (**a**) General block diagram; (**b**) Flow chart for baseband transmission experiment; and (**c**) Flow chart for passband transmission experiment.

For baseband experiments, the general measurement set-up block diagram displayed in Figure 5(a) was used. It consists of a function generator (Agilent, 33250A Function/Arbitrary waveform generator), a differential probe and an oscilloscope (Agilent, MSO6104A Mixed signal oscilloscope). As usual, all experiments done here have been performed carefully to take care of the common ground problem by using a differential probe (Agilent, 1141A) and battery-powered equipment. The flow chart is shown in Figure 5(b). During the experiment, the 500 Hz square wave with 50% duty-cycle (1 ms pulse width), peak-to-peak amplitude of 1 V from the function generator was applied to the cleansed human limb directly, and the received signals were detected via differential probe. The time domain signals were showed in the oscilloscope for recording. On the calculation side, given the transfer function, the calculated output signal in time-domain can be obtained by taking the Inverse Fourier Transform (IFT) of the product of the transfer function and the Fourier Transform (FT) of the input signal.

**Figure 6.**
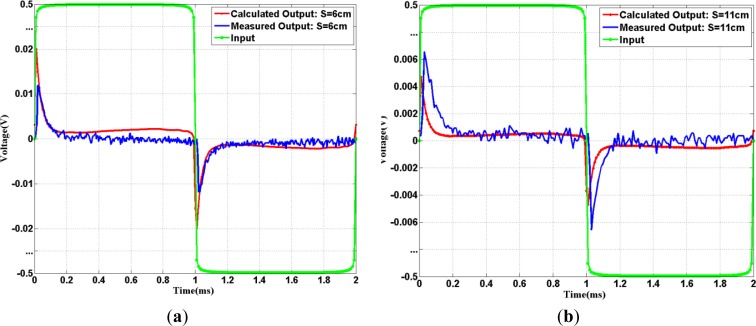
Input and Output of baseband IBC transmission for 500 Hz square wave: (**a**) S = 6 cm; (**b**) S = 11cm.

The corresponding output results for input square waves in baseband transmission in calculations and experiments are displayed in Figure 6. As shown in Figure 6(a,b), the calculated results are consistent with the measured results. Less attenuation transmissions occur near the time instants around 0 and 1 ms while large attenuation/distortion happens elsewhere. These spike-like outputs for pulse-input phenomena demonstrate that galvanic-type IBC channel for baseband transmission is very dispersive. The reason is that pulse amplitude changes in input lead to high frequency components, and high frequency components pass easier than lower frequency components as discussed in our previous Section. These non-flat frequency responses belong to a form of distortion that occurs when different frequencies are attenuated by different amounts, resulting to severe distortion for pulse transmissions. In order to quantify the output waveform distortion for the baseband input, the total harmonic distortion among the first four non-zero harmonics: (*THD*_4_) is defined as:

(11) $${\mathrm{THD}}_{4}=\frac{\sqrt{\sum_{2}^{4}\mathrm{non-zero}\;\mathrm{harmonic}\;\mathrm{powers}}}{\mathrm{fundamental}\;\mathrm{harmonic}\;\mathrm{voltage}}$$

The reason why we take only up to the 4th harmonics here is to have a fair comparison with passband transmission which will be discussed later in this Section. Like the 500 Hz square wave transmitted in the baseband channel, low frequency harmonics suffer higher attenuation, which will cause *THD*_4_ higher than that of ideal square wave around 41.4%. The calculation square wave *THD*_4_ result is around 96% while the corresponding measurement *THD*_4_ results are 99.7% and 99.2% for S = 6 cm and 11 cm, respectively. The *THD*_4_ deviation from the ideal case is around (55–58)%, which indicates signal transmitted via baseband technique in IBC is not suitable (pulse-shape → spike-shape).

**Figure 7.**
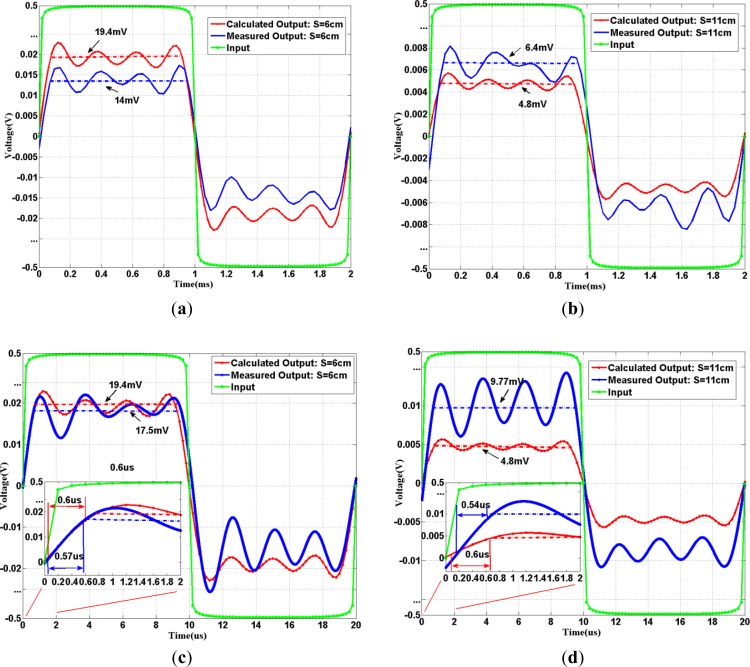
Input and Output (after demodulation) in passband IBC transmission for square wave: (**a**) 500 Hz square wave at S = 6 cm. (**b**) 500 Hz square wave at S = 11 cm. (**c**) 50 kHz square wave at S = 6 cm (**d**) 50 kHz square wave at S = 11 cm.

For passband experiments, two input square waves (one at 500 Hz, the other at 50 kHz) are modulated to the same carrier of 500 kHz. The 500 kHz carrier is chosen because it is located at around the center of low attenuation frequency band as shown in Figure 4. The general block diagram of passband experiment set-up is again shown in Figure 5(a), which consists of a vector signal generator (Agilent, N5182A MXG Vector Signal Generator), a differential probe and a signal analyzer (Agilent, N9020A MXA Signal Analyzer). The corresponding flow chart is displayed in Figure 5(c). During the experiment, the input square wave (500 Hz or 50 kHz) with 50% duty-cycle, peak to peak amplitude of 1 V, was modulated to high frequency band using BPSK with 500 kHz carrier by vector signal generator. The modulated signal was then applied to the cleansed human skin by the electrodes. The received signal was detected via differential probe and the demodulation was performed by the digital vector signal analysis software (Agilent, 89601A) on signal analyzer. During the demodulation process, the center frequency was set to 500 kHz, and the symbol rate was set to the original frequency of the square wave. However, the equipment software can only perform demodulation [32] on the limited bandwidth corresponding to the symbol rate. And the limited bandwidth contains only four non-zero harmonics of the square wave in our experiments. Therefore, in order to make fair comparison, we considered the similar situation in the calculation process by actually limiting the demodulation bandwidth of containing up to the first 4 non-zero harmonics, as indicated to Equation (11). Both calculation and measurement results of demodulation signals in passband transmission are shown in Figure 7.

As seen in Figure 7, the time-domain signal outputs basically retain the shape of input square pulses (compared with the sum of 1st four harmonics). As expected, there are always amplitude differences between the calculated and measured results. The amplitude deductions of the measured output signal for an extra 5-cm distance are around 46% and 55% for 500 Hz and 50 kHz, respectively. In the calculated result, the amplitude deduction is around 57% for 500 Hz and 50 kHz input. The differences between calculated and measured results mainly come from the dynamic physiological process of human body. The amplitudes of the calculated output waveforms are about 19.4 mV for both 500 Hz and 50 kHz input square waves at S = 6 cm; while the amplitudes of the calculated output waveforms are around 4.8 mV for S = 11 cm. The reason is that both input square waves were carried on the same the carrier frequency at 500 kHz, and the bandwidths of the two waves are within the relatively flat low-attenuation frequency band; hence they suffer the similar attenuation for a fixed separation S. The output pulses shown in Figure 7(a,b) have the *THD*_4_ around 41.9% in calculation and around 42.3% in measurement. Comparing with those values (upper ninety %) in baseband IBC transmissions, passband IBC transmission obtains lower distortion and has a much better communication performance. In Figure 7(c,d), the *THD*_4_ in measurement is around 42.4% and 41.7% at S = 6 cm and 11 cm, respectively. All passband *THD*_4_ are close to ideal square wave case, indicating rather low distortion for passband transmissions. In addition, all calculation and measurement rise times (defined as the time spent from 10% to 90% during transition) of output pulses (after demodulation) are around 0.6 μs at different distances, as shown in the insets of Figure 7(c,d).

From the relationship between rise-time and bandwidth, the corresponding system bandwidth would be inversely proportional to 0.6 μs. This indicates that system bandwidth is in the order of MHz, providing enough bandwidth for possible low distortion in passband transmission. Note that the maximum bandwidth of the transmitted signal is limited by the carrier. In summary, passband transmission with proper choice of carrier frequency would have rather good communication performance in terms of both attenuation and distortion.

## 4. Performance of Modulation Schemes over IBC Channel

The application of efficient digital modulation can improve power efficiency and bandwidth efficiency at a reasonable cost. From the results of the time domain and frequency domain analyses above, it is concluded that signal in passband transmission enjoys the benefit of lower attenuation and lower distortion than the baseband transmission counterpart. Therefore, we now evaluate and compare the performance of different modulation schemes with a carrier frequency of 500 kHz using the proposed IBC channel model. Moreover, the use of a carrier frequency at 500 kHz could be a balance between data rate and electromagnetic radiation.

Among the commonly available digital modulation techniques, FSK, PSK and QAM, high-ary modulation would not be considered due to either its high complexity in hardware, such as 16QAM, 16PSK, or due to its low bandwidth efficiency, such as 8FSK and 16FSK. PSK is generally superior than FSK in terms of bandwidth and power efficiency in the low-ary (≤8-ary) [33]. Therefore, we focus on the performance of the following three modulation schemes, namely, BPSK, QPSK, and 8PSK. Moreover, the data rate of the proposed IBC channel is limited by the bandwidth of the channel. It would be practical to consider low-ary power efficient modulation schemes since high data rate transmission is not practical in this case due to relatively low carrier frequency and high complexity in hardware.

**Figure 8.**
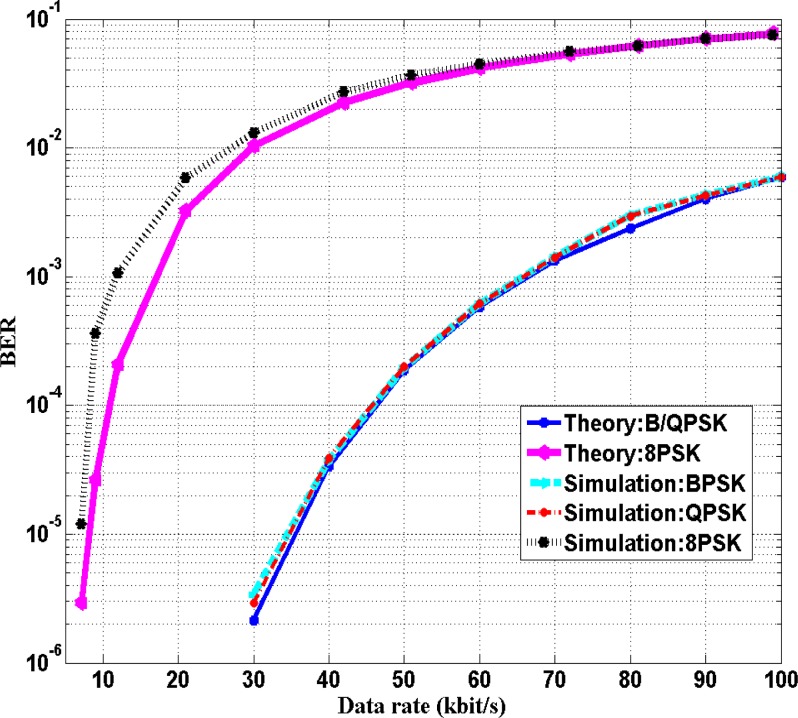
Performance of BPSK, QPSK and 8PSK *versus* data rate in simulation and theory at S = 11 cm.

**Figure 9.**
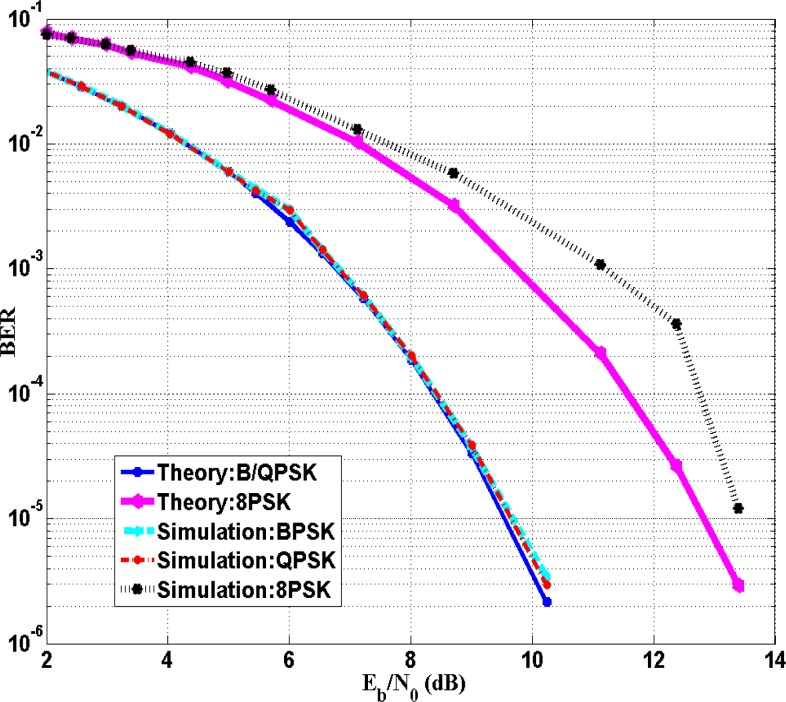
Performance of BPSK, QPSK and 8PSK *versus* SNR in simulation and theory at S = 11 cm.

The data symbols are first digitally modulated using BPSK, QPSK or 8PSK and then are pulse-shaped by the square root raised cosine filter with roll-off factor α = 0.5 before sending to the IBC channel. The noise within the human body mainly comes from various bioelectric signals, the environment and contact impedance between the electrodes and the human body. As the frequency ranges of bioelectric signals such as ECG, EMG and EEG signal are all lower than 10 kHz [34], therefore, noise contributed to the passband comes mainly from the contact between electrodes and human body, and other background noises. We assume that the noise is Additive White Gaussian Noise (AWGN) with power spectrum density (PSD) *N_0_*. From the measurements without applying input signal, the estimated value of background noise PSD *N*_0_ is found to be around −123 dBm/Hz. The transmit power for the transmitter was set to −25 dBm for a compromise between feasible calculation time and bit-error-rate (BER). The communication performance (BER) in simulation and theoretical calculation is compared. In the simulation, the channel characterized by transfer function (7) and AWGN noise with PSD −123 *dBm*/Hz is built, then a large amount (up to 10^7^ bits) of random bit stream with different bit rate and different modulation scheme is applied into this channel, and output bit-stream (with possible error bits) is obtained at the receiver after demodulation. The BER can be calculated based on the comparison between input and received data bits. In the theoretical calculation, the equivalent signal to noise ratio (*i.e.*, the ratio of average signal energy per bit to noise power spectral density *E_b_*/*N*_0_) can be calculated before demodulation at the receiver using the transmit power, the transfer function attenuation, *N*_0_ and data symbol rates. The theoretical BER can be calculated by the following equation:

(12) $$\mathrm{BER}=\{\begin{array}{ll} Q\left(\sqrt{2E_{b}/N_{0}}\right) & \mathrm{for}\;\mathrm{BPSK}\;\mathrm{and}\;\mathrm{QPSK}\\ Q\left[\text{sin}(\pi /8)\times \sqrt{6E_{b}/N_{0}}\right] & \mathrm{for}\;8\;\mathrm{PSK} \end{array}$$

where Q(*x*) is the Gaussian Q-function. At a transmission distance of 11 cm, Figure 8 shows the BER performances of BPSK, QPSK and 8PSK for the data rates between 6 kbps and 100 kbps and the BER performances *versus E_b_*/*N*_0_ are presented in Figure 9. As depicted in Figures 8 and 9, the performance of BPSK and QPSK are similar. The BER is lower than 10^−3^ when *E_b_*/*N*_0_ is higher than 6.5 dB with the maximum bit rate of 70 kbps for both BPSK and QPSK modulation. For 8PSK, BER is lower than 0.01 when *E_b_*/*N*_0_ is higher than 7 dB with the bit rate lower than 30 kbps. Among the three modulation methods, 8PSK obtains the highest bandwidth efficiency; while QPSK is twice as high as that of BPSK. However, the 8PSK suffers high BER at the same bit rate. In other words, 8PSK trades bandwidth efficiency off BER while QPSK obtains lowest BER with higher bandwidth efficiency. Thus, considering the bandwidth efficiency and BER, QPSK modulation scheme obtains the highest bit rate with low BER and is the optimal modulation scheme among BPSK, QPSK and 8PSK.

## 5. Conclusions

In this paper, we have investigated the channel characteristics of a galvanic-type IBC based on the transfer function derived from our analytical quasi-static EM model. Both time and frequency domain experimental results evidence good agreements with the model calculations, which demonstrate the benefits of using an analytical transfer function in analyzing IBC channel characteristics. The high pass channel characteristic suggests that a galvanic-type IBC can be suitably operated at least in hundreds of kHz of carrier for lower attenuation in sub-MHz frequency region and maintain reasonably good data rates. Moreover, the harmonic distortion in the IBC channel time domain analyses is around 96% for baseband transmission with the 500 Hz input square wave; while it reduces to around 41.9% for passband transmission, close to the ideal case of considering equipment ability of four harmonics. The results suggest that passband transmission can achieve low distortion even without channel equalization. Considering power efficiency, bandwidth efficiency and hardware complexity through theoretical calculation and simulation, QPSK is the optimum modulation method among BPSK, QPSK and 8PSK modulation schemes for reasonable BER at the low transmitted power of −25 dBm in passband transmission. The calculation, experimental and simulation results in this work have provided useful information for transmission method and modulation scheme selection in the IBC system design. In the future, we will continue to research on the optimum IBC method in terms of power, bandwidth and cost.
